# Progress in pediatrics in 2015: choices in allergy, endocrinology, gastroenterology, genetics, haematology, infectious diseases, neonatology, nephrology, neurology, nutrition, oncology and pulmonology

**DOI:** 10.1186/s13052-016-0288-x

**Published:** 2016-08-27

**Authors:** Carlo Caffarelli, Francesca Santamaria, Dora Di Mauro, Carla Mastrorilli, Virginia Mirra, Sergio Bernasconi

**Affiliations:** 1 Clinica Pediatrica, Department of Clinical and Experimental Medicine, Azienda Ospedaliera-Universitaria, University of Parma, Parma, Italy; 2 Department of Translational Medical Sciences, Federico II University, Naples, Italy; 3 Pediatrics Honorary Member University Faculty, G D’Annunzio University of Chieti-Pescara, Chieti, Italy

**Keywords:** Allergy, Endocrinology, Gastroenterology, Genetics, Onco-haematology, Infections, Neonatology, Neurology, Nutrition and pulmonology

## Abstract

This review focuses key advances in different pediatric fields that were published in Italian Journal of Pediatrics and in international journals in 2015. Weaning studies continue to show promise for preventing food allergy. New diagnostic tools are available for identifying the allergic origin of allergic-like symptoms. Advances have been reported in obesity, short stature and autoimmune endocrine disorders. New molecules are offered to reduce weight gain and insulin-resistance in obese children. Regional investigations may provide suggestions for preventing short stature. Epidemiological studies have evidenced the high incidence of Graves’ disease and Hashimoto’s thyroiditis in patients with Down syndrome. Documentation of novel risk factors for celiac disease are of use to develop strategies for prevention in the population at-risk. Diagnostic criteria for non-celiac gluten sensitivity have been reported. Negative effect on nervous system development of the supernumerary X chromosome in Klinefelter syndrome has emerged. Improvements have been made in understanding rare diseases such as Rubinstein-Taybi syndrome. Eltrombopag is an effective therapy for immune trombocytopenia. Children with sickle-cell anemia are at risk for nocturnal enuresis. Invasive diseases caused by *Streptococcus pyogenes* are still common despite of vaccination. No difference in frequency of antibiotic prescriptions for acute otitis media between before the publication of the national guideline and after has been found. The importance of timing of iron administration in low birth weight infants, the effect of probiotics for preventing necrotising enterocolitis and perspectives for managing jaundice and cholestasis in neonates have been highlighted. New strategies have been developed to reduce the risk for relapse in nephrotic syndrome including prednisolone during upper respiratory infection. Insights into the pathophysiology of cerebral palsy, arterial ischemic stroke and acute encephalitis may drive advances in treatment. Recommendations on breastfeeding and complementary feeding have been updated. Novel treatments for rhabdomyosarcoma should be considered for paediatric patients. Control of risk factors for bronchiolitis and administration of pavilizumab for preventing respiratory syncytial virus infection may reduce hospitalization. Identification of risk factors for hospitalization in children with wheezing can improve the management of this disease. Deletions or mutations in genes encoding proteins for surfactant function may cause diffuse lung disease.

## Background

This review highlights main advances in allergy, endocrinology, gastroenterology, genetics, haematology, infectious diseases, neonatology, nephrology, neurology, nutrition, oncology and pulmonology in childhood. Papers were primarily selected from the most accessed articles published in Italian Journal of Pediatrics and in international journals in 2015.

## Review

### Allergy

Food allergy is a frequent public health complaint [1–3] with potentially life threatening reactions. Several observational studies have suggested that early introduction of certain solid foods in infants may be associated with a decline of food allergy. Interventional studies were developed to more definitively ascertain these outcomes. In infants with atopic eczema, egg introduction at 4 months resulted in a nonsignificant lower rate of egg allergy at 12 months of age in comparison with a control group who went on egg-free diet [2]. Du Toit et al. [1] introduced peanut at a median age of 7.8 months, in children at risk for peanut allergy because of atopic eczema o egg sensitization. They found that peanut consumption was effective in reducing the prevalence of peanut allergy both in children who were not sensitized to peanuts (primary prevention) and in those who were sensitized (secondary prevention) in comparison with controls. Consequently, a consensus communication [4] recommended early introduction of peanut to prevent peanut allergy in children at risk. However, the term early seems to be inappropriate and it may be misinterpreted, since findings by Du Toit et al. [1] do not imply that current weaning plan should be modified [5]. Findings of interventional studies on inducing food tolerance by the oral route raise the question whether cutaneous exposure to large amounts of food allergens [6] or inhalation of food particles [7] may be also used to prevent food allergy.

In the pediatric population, allergic-like symptoms are one of the most common causes for consultation in a primary care setting. Oral food challenge remains the gold standard for diagnosis of food allergy. This happens also when other concomitant precipitating factors are required to trigger an allergic reaction [8]. Regarding other investigations, Caglayan et al. [9] showed that atopy patch test for the evaluation of egg and cow’s milk allergy should not be routinely used. Fiocchi et al. [10] aimed to clarify the sensitization rate in children with common allergic symptoms. They determined serum specific IgE levels to foods and inhalants in 532 children (<15 years of age) with at least one allergy-like symptom, from 21 primary care centers in two geographic areas of Italy and Spain. They found 267 (50.2 %) atopic children with at least one positive serum specific IgE antibody. Multiple sensitizations were common: 14 were monosensitized, 37 were sensitized to 2–3 allergens and 49 % to more than 3 allergens. This point out that molecular diagnosis would be often necessary to distinguish between polysensitized children who are sensitized to genuine allergens and those sensitized to cross-reactive allergens [11]. The average number of symptoms in the atopic group was 3.3 vs 2.8 in the non-atopic group. The prevalence of sensitization to single allergens was highest for grass, ragweed and house-dust mites (19–28) and among tree allergens was for olive trees (16.5 %). Cow’s milk and egg white were the most sensitizing foods. Food allergen sensitization predominated in younger children whereas the inverse occurred with inhalant allergens. A significant positive correlation between patient age and number of sensitizations was found. This study highlights that more specific means than symptoms alone, are needed to reduce unnecessary specific IgE measurement in primary care. However, it should be also taken into account that when measurement of serum specific IgE levels and not skin prick tests to allergen extracts are performed, some allergic sensitizations may be missed. Along this line, it has been recently shown that this happens also with molecular allergens. Asero et al. [12] found that results of specific IgE to Phl p 12 (grass profilin) and skin prick tests to natural profilin (Pho d 2), purified from date palm extract gave a concordant response in 91 % of cases. In asthmatic patients, exhaled breath biomarkers [13] may be useful for identifying allergic patients. Among them fractional exhaled nitric oxide (FeNO) is significantly correlated with specific IgE antibodies levels to house dust mite in atopic asthmatic patients [14]. Furthermore, FeNO values greater that 21 ppb are associated with airway eosinophilia in corticosteroid-naïve patients [15] and are helpful in predicting loss of asthma control [16]. It has been found increased levels of oxidative stress markers such as pH [17] and hydrogen peroxide [18], that are sometimes related to decrease lung function in asthmatics. Hydrogen peroxide induces apoptosis of eosinophils and enhances the resolution of allergic inflammation [19]. Longitudinal studies are warranted to establish whether such markers may be helpful in diagnosis and control of asthma. Exercise induced bronchoconstriction (EIB) is commonly experienced by asthmatic children. Johansson et al. [20] showed that the prevalence of EIB was 19.2 % in a general population of adolescents and 42.4 %. In subjects with exercise-induced dyspnea, less than 50 % of subjects with EIB had ever had asthma. In agreement, it has been shown that EIB is a feature of not only of asthma but also of allergic rhinitis and atopic eczema [21]. Furthermore, 10.8 % of subjects with exercise-induced dyspnea presented exercise-induced laryngeal obstruction. This can be an important differential diagnosis in adolescents breathing problems during exercise [20].

### Endocrinology

Advances have been reported in obesity, short stature and autoimmune endocrine disorders. Childhood obesity and overweight have increased worldwide during the last decades. Lobstein et al. [22] highlight the need for obesity prevention programme in high-income countries. Comprehensive interventions rather than single-component interventions, focused on meals, classroom activities, sports, and play activities are essential to reverse the obesity trend. Interventions should be conducted at kindergarten, school, home, community. Childhood obesity prevention interventions have been demonstrated to be cost effective. Another question is how early prevention programme should start. The NOURISH randomized controlled trial [23] showed that in infancy, complementary feeding practices promoting self-regulation of intake and preference of healthy foods had effects that were sustained up to 5 years of age. However, no effect was noted for anthropometric measures or the prevalence of overweight/obesity. Obesity prevention should take into account the industrial interest in creating overweight [22]. So, regulatory steps should be taken to protect children from exposure to advertising that may influence dietary preferences. Specific nutrient standards for products are required for improving nutrition. Children and adolescents who are obese have risk factors for cardiovascular disease, prediabetes, bone and joint problems, sleep apnea, and social and psychological problems and to be obese as adults. Little is known whether there are subgroups of obese children who are at greater risk for developing cardiovascular diseases. Chang et al. [24] studied lipid profile, insulin, glucose, leptin and circulating levels of inflammatory markers, such as tumor necrosis factor-α, interleukin-6, monocyte chemoattractant protein-1, and the high-sensitive C-reactive protein, in male children, 19 obese, 10 overweight and 16 controls. Obese and overweight subjects had insulin resistance, significant higher inflammatory cytokine levels including high-sensitive C-reactive protein, PAI-1, tumor necrosis factor-α and leptin levels. Lipid profile was abnormal only in obese children. A factor analysis identified three domains that explained 74.08 % of the total variance among the obese children (factor 1:lipid, factor 2: obesity inflammation, factor 3: insulin sensitivity domains). These domains might be applied to predict the onset of cardiovascular diseases in adulthood. The treatment of childhood obesity is time consuming, problematic, and costly. Two studies addressed the role of various dietary interventions and physical activity in children. Stagi et al. [25] assessed the effect of various treatments on body mass index (BMI) and basal insulin levels in obese children and adolescents with family history of obesity and type 2 diabetes mellitus (T2DM). In a randomised study, 133 children were divided into three arms. Arm A was treated with low glycaemic index diet and new complex of polysaccharidic macromolecules (cellulose, hemicellulose, pectin, mucilages) that slows down the rate of carbohydrate and fat absorption. Arm B was treated with low glycaemic index diet diet and Arm C treated with energy restricted diets. After 1 year, BMI reduction was significant in individuals subjected to low glycaemic index diet but not to energy restricted diets diet. Furthermore, Arm A subjects have significantly reduced BMI-SDSs compared to Arm B and Arm C subjects. Insulin and glucose levels were significantly reduced in Arm A, whereas in Arm B, we observed a significant reduction in insulin but not in glucose levels. In Arm C, insulin and glucose levels did not differ from baseline. The benefit of a complex of polysaccharidic macromolecules (cellulose, hemicellulose, pectin, mucilages) added to low glycaemic index diet should be confirmed with further studies that include larger sample with longer follow-up.

Verduci et al. [26] evaluated whether a 1-year intervention based on normocaloric diet and physical activity may impact the BMI status, blood lipid profile and glucose metabolism indicators in 90 obese children. At the end of intervention, children showed a reduction in BMI z-score, triglycerides and triglyceride glucose index, and an increase in HDL cholesterol. Prevalence of insulin resistance declined from 51.8 to 36.5 and prevalence of metabolic syndrome from 17.1 % to 4.9 %.

Several insights on short stature have been reported. National studies on the prevalence of short stature are important. However, the prevalence of short stature may have significant regional variations in relation to race, climate, psychosocial problems and malnutrition. Wang et al. [27] assessed height in 12,009 students aged from 7 to 18 years, in Anhui province (China). Short stature was defined according to the criteria of the Chinese Medical Association as follows: height under 2SD of average height in same race, gender and age; height below the third percentile of average height in same race, gender and age. The prevalence of short stature was 3.16 % and it was similar to that reported in other regions of China. The rate was higher in rural areas than in urban areas, higher in economically backward areas than in economically developed areas. They concluded that a local government plan is needed to prevent short stature. It should carry out measures such as health education, reasonable diet, fitting exercise, adequate sleep and improving life style. Among children with idiopathic short stature effective treatment modalities are still under evaluation. Recombinant growth hormone (rGH) or insulin-like growth factor-1 (rIGF-1) can be assumed as an alternative in the treatment of these children. Recently, rGH has been approved for therapy in idiopathic short stature but reports on this treatment are limited. Siklar et al. [28] recruited 21 ISS children of unknown etiology and with IGF-1 levels below the −2 SDs of normal levels with the aim of determining short and long-term effects of rGH treatment. The height of the patients improved from −3.16 ± 0.46 SD score (SDS) to −1.9 ± 0.66 SDS. At the end of the follow-up period (5.42 ± 1.67-year), mean height SDS was −1.72. Almost 40 % of patients reached their target height. A female preponderance was noted in the responder group. The rGH treatment was safe.

Response to GH therapy is variable in subjects and depends on individual factors including age, BMI, genetic and gender. The exon 3-deleted GH receptor (GHRd3) has been showed to be associated with better growth response to r-GH treatment in children with idiopathic short stature and children born small for gestational age. Valsesia et al. [29] analyzed the clinical and genetic data generated in the PREDICT study and in the PREDICT long-term follow-up study to investigate association between GHRd3 and growth response to r-hGH over 3 years in relation to severity of GH deficiency. GHRd3 carriers with higher peak GH level had better growth (C2.7 cm; C0.2 SDS) than those with low peak GH level. Similar patterns were observed for GH-dependent biomarkers. Gene expression profiles were significantly different between groups, indicating that the interaction between GH status and GHRd3 carriage can be identified at a transcriptomic level. GH treatment is frequently prescribed in Prader Willi Syndrome (PWS) at early infancy or childhood and leads to improve changes in body composition, physical activity and growth velocity. Butler et al. [30] provided new growth charts of weight, height, head circumference and BMI for non-GH treated subject with PWS aged 3 to 18 years to be used in clinical setting. Anthropometric measures were obtained from 120 PWS subjects and standardized growth charts representing 7 percentile ranges were developed along with the normative third, 50th, and 97th percentiles from national and international data. PSW-specific growth standards must be used in subjects with PWS to monitor growth patterns and nutritional status and plan individual medical care, diet intervention and physical activities.

Regarding autoimmune endocrine disorders disease, only few reports have found that in childhood, Graves’ disease (GD) and Hashimoto’s thyroiditis (HT) may follow one another in the same individuals. These autoimmune thyroid disorders are more frequent in patients with Turner syndrome or Down syndrome. Aversa et al. [31] retrospectively analysed the sequential phenotypic conversion from HT to GD and the subsequent evolution of GD in a series of 12 children and adolescents with Down syndrome. All were prepubertal except one. Patients with HT diagnosis differed in thyroid hormone levels and in use of L-thyroxine. Time interval between HT diagnosis and GD onset ranged from 0.7 to 6.5 years (median 4.2). There was no correlation between duration of L-thyroxine treatment and time interval HT – GD. After metimazole withdrawal, one third of patients exhibited a spontaneous change of thyroid function, from hyperthyroidism to hypothyroidism and needed L-thyroxine treatment. These children did not significantly differ from the other ones in thyroid function tests, serum autoantibody levels at HT diagnosis or in thyrotropin receptor autoantibodies values at GD presentation. These data indicate that children with Down syndrome might manifest over time a phenotypic metamorphosis from HT to GD and they may subsequently fluctuate from hyperthyroidism to hypothyroidism.

The association of autoimmune thyroid disease with autoimmune type 1 diabetes mellitus is well established [32]. Balsamo et al. [33] enrolled 152 children and adolescents at type 1 diabetes mellitus onset who had positive antithyroid antibody in 11 % of cases. They observed that thyroid function at type 1 diabetes mellitus onset is impaired mainly in association with metabolic derangement, irrespective of thyroid autoimmunity. Patients with antithyroid antibodies at type 1 diabetes mellitus onset were at higher risk of developing hypothyroidism over time.

### Gastroenterology

Several studies on celiac disease (CD) and non-celiac gluten sensitivity have added insights into pathophysiology and treatment. Sarno et al. [34] recognized that according to family studies, 87 % of celiac disease (CD) heritability could be explained. Among genetic factors associated with CD, they described risk loci, including genetic human leukocyte antigen HLA class II region, HLA-DQ2 or HLA-DQ8 haplotypes, major histocompatibility complex class I region and non-HLA genes. They reviewed several studies that evaluated the role of environmental factors for triggering CD in the population with genetic predisposition. They noted that neither breastfeeding nor timing of gluten introduction reduced the risk for CD development. They point out that CD risk might be reduced by avoidance of high amounts of gluten in the first year of life [35]. Finally early infections, especially due to adenovirus and rotavirus, may have an impact on the risk of CD by inducing inflammatory T cell responses. Future studies on reduction of immunogenic gluten and vaccination may be helpful to develop an effective prevention of CD. Non-celiac gluten sensitivity [36, 37] is a recently documented syndrome caused by ingestion of gluten whose symptoms should be mainly distinguished from irritable bowel syndrome [38]. A consensus conference [39] has proposed new criteria for identifying non-celiac gluten sensitivity after excluding celiac disease or allergy to wheat. Non-celiac gluten sensitivity should be suspected when there is a decrease of >30 % from the baseline score of a standardized questionnaire for at least 50 % of a 6-week strict gluten-free diet. The definitive mean for the diagnosis is a positive double-blind placebo-controlled challenge. The challenge consists in a 1-week gluten diet (8 g) followed by a 1-week gluten-free diet and then a second 1-week challenge. Response to challenge should be assessed by the questionnaire whose results are positive when there is >30 % score variation between the gluten and the placebo challenge. As recognized by Authors [39], the threshold of 30 % increment in symptoms needs to be validated.

### Genetics

Sex chromosomal aneuploidies in males are rare diseases with an overwhelming involvement of endocrinological and auxological issues. The most common of them is Klinefelter syndrome (KS) [40]. Brain magnetic resonance imaging (MRI) studies on KS have provided evidence that sex-chromosome polysomy exerts specific effects on brain development. Milani et al. [41] described a patient with a 48,XXXY/49,XXXXY mosaicism who showed some unusual neuroradiological features. The patient is a 20-months-old boy, who at birth had facial dysmorphysms, inverted nipples, and ventricular septal defect. He was first visited because of developmental delay, congenital heart defect and hypogenitalism. An MRI showed an asymmetric dysmorphic appearance of the posterior cranial fossa, that had small size, with dysmorphism of cranio-cervical junction and reduced visualization of cerebrospinal fluid spaces, that were confirmed at the computerized tomography. Brain abnormalities are frequently found in KS variants [42, 43], but also neurocognitive abnormalities, like as auditory and motor systems that often in KS are selectively affected [44]. Therefore, the supernumerary X chromosome seems to have a negative effect on white matter and central nervous system development. The patient described is the first case of a cranio-cervical junction malformation associated with 48,XXXY/ 49,XXXXY syndrome. It is important to underline that skeletal abnormalities are not exclusively related to the limbs, but also to the axial structures (such as the cervical spine), thus suggesting that the neuroradiological assessment is potentially useful in the diagnostic approach to patients with 48,XXXY and 49,XXXXY syndrome. Rubinstein-Taybi syndrome (RSTS) is an extremely rare multiple congenital anomaly/intellectual disability syndrome, with an estimated prevalence of one case per 125,000 live births. Given the complexity and rarity of RSTS, there are still numerous unanswered questions about it. In this review Milani et al. summarized the clinical features and genetic basis of RSTS, and highlighted the areas for future studies on appropriate diagnostic protocol and follow-up care for RSTS [45]. RSTS is characterized by slow development of height and weight, microcephaly, dysmorphic facial features, broad thumbs, and big toes. Over 90 % of individuals survive to adulthood, and healthcare for these patients is particularly complex, time-consuming, and often not standardized in specific guidelines. The gene most frequently involved is CREBBP [46]. Alterations in the E1A-binding protein p300 have also been detected, but many cases have been diagnosed only on a clinical basis [47]. Little is known about genotype-phenotype correlations in RSTS. Recurrent mutations maybe a key tool in addressing genotype-phenotype correlations in patients sharing the same defects and specific clinical signs, as demonstrated in two cases in a clinical cohort of 46 RSTS patients [48]. Nevertheless, a severe phenotype has been reported in RSTS patients who show large gene deletions. Novel genetic and epigenetic therapies may be promising, but there is still an urgent need to improve and personalize the standard follow up protocol [49]. In order to improve it, the authors drafted their follow-up proposal.

### Haematology

Children with immune trombocytopenia and sickle-cell anemia (SCA) have been intensively investigated. Childhood immune thrombocytopenia is frequently a self-limiting disease, however in 13–36 % of cases it lasts more than 12 months and it is classified as chronic. Current treatments include immunosuppressors, such as corticosteroids and rituximab, immunoglobulins, and splenectomy, with high side-effects and potential short-term and long-term risks. Eltrombopag is an oral non-peptide thrombopoietin receptor agonist that binds to the transmembrane domain of the thrombopoietin receptor, leading to signal transduction through various pathways, including Jak/STAT and MAPK, which results in proliferation and differentiation of megakaryocytes and increased platelet production. Eltrombopag is an effective therapy for chronic immune thrombocytopenia, severe aplastic anemia, and chronic hepatitis C-associated thrombocytopenia in adults. The effect of eltrombopag in children with chronic immune thrombocytopenia have been investigated by two parallel randomised, multicentre, placebo controlled trials, PETIT trial [50], as a phase 2 trial, and PETIT2 study [51] as the phase 3 trial. In PETIT study, eltrombopag produced a sustained platelet response of 50x10^9^ per L in 62 % of patients vs 32 % in placebo group, from week 1 to week 6; in PETIT2 study, in 40 % of children vs 3 % in placebo group, in the double-blind period of 13 weeks. During the open-label phase, about 80 % of patients achieved a platelet count of 50 × 10^9^ per L or more at least once in both studies. Reduction in bleeding was also observed. Adverse events were mostly mild and serious adverse events (reversible increased alanine aminotransferase concentrations, pneumonia, aseptic meningitis, neutropenia, and anemia) were infrequent. At variance from adults, no malignancy and thrombosis occurred. Further data on cost, availability of more effective options, assessment of patient-reported outcomes and patient adherence to long-term daily medication are warranted to understand whether eltrombopag is a suitable therapeutic option for children with chronic immune thrombocytopenia in clinical practice [52].

Children with SCA are prone to sleep pathology because of smaller bladder capacity during sleep, hypostenuria, increased arousal thresholds, sleep-disordered breathing (obstructive sleep apnea syndrome). Mascarenhas et al. [53] performed a retrospective study comparing polysomnography results in 65 children with SCA versus 65 children without SCA with suspected OSAS. Mean SpO_2_ and minimum SpO_2_ were significantly lower in SCA patients. No significant difference was found between groups in efficiency, latency and percentage of sleep phases. SCA group was at higher risk for nocturnal enuresis (35.4 % vs 6.2 %, *p* < 0.01). Eneh et al. [54] performed a questionnaire-based, case–control study evaluating nocturnal enuresis and possible risk factors among 70 children, aged 5 to 11 years, with SCA and 70 age- and sex-matched controls. Although, there was no difference in nocturnal enuresis between the 2 groups, the prevalence of nocturnal enuresis was significantly higher in males with SCA than in male controls and in subjects whose parents had a childhood history of enuresis. They found no relationship with socio-economic status. Their findings show that reduced responsiveness to toilet training in boys and familiar predisposition may play a role in nocturnal enuresis. This study point out that school children with SCA should be frequently evaluated for nocturnal enuresis, particularly if they are males and have parental history of nocturnal enuresis in childhood.

### Infectious diseases

Epidemiologic studies continue to document changes in the post-vaccination era. In the United States MacNeil et al. [55] found that *Neisseria meningitidis* remains an important cause of infectious disease in children <1 year, despite the use of pneumococcal vaccines in infants. They collected data from active, population- and laboratory-based surveillance, including cultures from blood, CSF, joint or pleural fluid for *N. meningitidis*, during 2006 through 2012. They estimated 113 cases annually of meningococcal disease among infants aged <1 year, for an overall incidence of 2.74 per 100 000 infants and a death rate of 6 children per year. Serogroup B was responsible for 64 % of cases, followed by serogroup Y and C responsible for 16 and 12 % of infant cases respectively. Authors suggest that future meningococcal disease vaccination strategies should be targeted on serogroup B meningococcal disease. Furthermore, they may include a maternal vaccination program to protect infants aged <1 year as they would be too young to have received the minimum 2 or 3 doses of vaccine that are needed to prevent the disease.

Azzari et al. [56] studied the burden of bacteremia and invasive disease (ID) in 920 children less than 5 years old with a fever of 39 °C or greater in a prospective, multi-centre, hospital-based study. They found ID in 225 children, sepsis in 38 and non-invasive disease in 629. Twenty-two (9.8) children with ID, 2 (5.3) with sepsis and 10 (1.6 %) with a clinical diagnosis of non-invasive disease were bacteremic, having at least one positive sample, detected either by molecular assessment (PCR), culture, or both. In children with bacteremia, the most common diagnoses were community-acquired pneumonia (15/34), pleural effusion (4/34) and meningitis (4/34). It was noted a higher sensitivity of molecular versus cultural techniques for detecting bacteremia. Among bacteremic cases, *Streptococcus pneumoniae* was detected in 85.3 % (29/34) of cases, *Haemophilus influenzae* in 3 (2 non-typeable and 1 capsulated), *Escherichia Coli* in 1, and *Neisseria meningitidis* in 1. The most commonly detected *Streptococcus pneumoniae* serotype was 19A, detected in four cases. Community-acquired pneumonia was due to *Streptococcus pneumoniae* in 14 children, 3 due to serotype 3 and 3 due to serotype 14. Pleural effusion were always due to *Streptococcus pneumoniae*, meningitis were due to *Streptococcus pneumoniae* in 3 patients and to *Neisseria meningitides* in 1. The mean direct medical cost of bacteremic cases was 3306 euro. This study confirms that *Streptococcus pyogenes* is an important global pathogen, causing considerable morbidity in the paediatric population with high costs and it indicates the need for preventing pneumococcal infection by vaccination.

In the past, the belief that measles, mumps and rubella vaccination may cause autism had induced some parents to not immunize their children. Using an administrative claims database associated with a large US health plan, Jain et al. [57] confirmed that measles, mumps and rubella vaccine was not associated with an increased risk of autistic spectrum disorder at any age in 95 727 children. They found that children with an older sibling with autistic spectrum disorder, more frequently develop autistic spectrum disorder, compared with those with siblings without autistic spectrum disorder (*p* < .001). It is reassuring that families with a child already affected by autism were not less likely to have younger children vaccinated.

Numerous studies are evaluating measures to prevent the risk of infectious diseases. In low-income countries, Rotavirus disease occurs at a younger age than in high-income countries and in regions with a high burden of Rotavirus disease the success of Rotavirus vaccine is often suboptimal. Bines et al. [58] showed that coverage and efficacy of oral RV3-BB rotavirus vaccine derived from newborn with asymptomatic rotavirus infection are improved by a dose of rotavirus vaccine given at birth. In a phase 2a randomized, double-blind, three-arm, placebo-controlled trial, participants were randomized to oral RV3-BB rotavirus vaccine with the first dose given at 0–5 days after birth (neonatal schedule, 30 participants), to vaccine with the first dose given at about 8 weeks of age (infant schedule, 27 participants), or to placebo (32 participants). After randomization children received four oral doses of product at around 8 week intervals. The primary outcome was cumulative vaccine take (a serum immune response of anti-rotavirus IgA or serum neutralizing antibodies or detection of RV3-BB virus in stool after administration of vaccine or placebo) after three doses of RV3-BB. The RV3-BB rotavirus vaccine was found to be immunogenic with a positive cumulative vaccine take detected in 90 and 93 % of participants after three doses in a neonatal or an infant schedule, respectively, and well tolerated. Acute otitis media (AOM) in children can be managed without antibiotics. However, it is the most common reason for their use. Overusing antibiotic may increase serious side effects, antibiotic resistance and costs. *Streptococcus salivarius* 24SMB is able to produce bacteriocin-like substances with significant activity against AOM pathogens [59]. Marchisio et al. [60] investigated the efficacy of *Streptococcus salivarius* 24SMB nasal spray in preventing AOM in 100 otitis-prone children, aged 1–5 years, with recurrent AOM in a randomized, double-blind, placebo controlled trial. Children received intranasal *S. salivarius* 24SMB or placebo twice daily for 5 days each month for 3 consecutive months. When enrolled, the children were free of AOM. They were initially treated with amoxicillin-clavulanic acid for 10 days, to facilitate *S. salivarius* 24SMB colonization. During the study, when an AOM were diagnosed it was treated with amoxicillin plus clavulanic acid for 10 days. In the group treated with *S. salivarius* 24SMB, fewer children experienced any AOM in comparison with those in the placebo group (30.0 vs 14.9 %; *p* = 0.076). The number of children colonized by *S. salivarius* 24SMB who experienced any AOM was significantly lower compared with the children who were not colonized. The nasal administration of *S. salivarius* 24SMB was safe and well tolerated. In clinical practice, limitations of the study [61] include use of amoxicillin-clavulanic acid as first line antibiotic in AOM instead of amoxicillin and treatment of all enrolled children with an amoxicillin-clavulanic acid even if they were healthy at baseline. This can lead to an increasing antibiotic resistance and it is in contrast with a 2010 Italian pediatric guideline for the treatment of AOM [62] that recommended a “watchful waiting” approach for children with AOM to decrease the use of antibiotic. Unfortunately, watchful waiting strategy is still far from routine use and the antibiotic prescription rate continues to be high. Palma et al. [63] found no difference in the frequency of antibiotic prescriptions between before the publication of the guideline and after, both in a whole population of 4,573 children with AOM seen at the Pediatric Emergency Department (82 % versus 81 %) and in all age classes. The most frequently prescribed antibiotic was amoxicillin-clavulanic acid (51 %) and its prescription rate was similar before and after guideline publication.

### Neonatology

Infants admitted to neonatal intensive care units (NICU), especially extremely low birth weight infants often receive an empirical antibiotic treatment, even if proven sepsis is diagnosed in a minority of cases [64]. Tzialla et al. [65] underline that unnecessary empirical administration of broad-spectrum antibiotics should be limited since it is associated with increasing multi-drug resistant bacterial infections, alteration of gut colonization which may lead to necrotizing enterocolitis (NEC) and increasing risk of invasive candidiasis. They also summarize current knowledge on the appropriate choice of antimicrobial agents and optimal duration of therapy in newborns with suspected or culture-proven sepsis. In the absence of randomized controlled trials that can definitely prove the best choice of antibiotics, an association of a penicillin or semisynthetic penicillin together with an aminoglycoside can be considered the best empirical regimen for early-onset sepsis [66]. Antistaphylococcal penicillin (oxacillin, flucloxacillin) plus an aminoglycoside should be the best option for late-onset sepsis (LOS); vancomycin should be given when infection by methicillin-resistant *Staphylococcus aureus* or coagulase-negative *Staphylococcus* is proven. Diagnosis of late-onset neonatal sepsis (LOS) can be difficult because clinical manifestations are not specific and none of the available laboratory tests can be considered an ideal marker. Poggi et al. [67] showed that presepsin (sCD14-ST) (P-SEP) may be an accurate marker for identifying LOS in preterm newborns. Presepsin is a soluble N-terminal fragment of the cluster of differentiation marker protein CD14, which is released into the circulation during monocyte activation upon the recognition of lipopolysaccharide from infectious agents. A prospective single-centre study on newborns <32 weeks’ gestational age who developed possible LOS was conducted. Blood samples for culture and count and for measuring P-SEP, procalcitonin, CRP were taken at enrolment (T0) and 1 (T1), 3 (T3), and 5 (T5) days after the first sample. P -SEP values were significantly higher in the LOS group (*n* = 21) than in the control group (*n* = 19) at any time. P-SEP achieved the best accuracy for prediction of probable sepsis at the cut-off of 885 ng/L with 94 sensitivity (95 CI 74–100) and 100 % specificity (95 % CI 84–100). Larger studies are required to confirm the role of P-SEP in early diagnosis of sepsis.

NEC is a multifactorial disease associated with prematurity. Use of broad-spectrum antimicrobials and dysbiosis [68, 69], may play a role in NEC onset. Therefore, interest is growing in preventing NEC by given oral probiotics [70]. Along this line, in a meta-analysis Aceti et al. [71] selected twenty-six studies on the effect of probiotics for preventing NEC in preterm infants. The majority of studies had severe or moderate flaws. However, they found that probiotics may be effective in preterm and in very-low-birth-weight infants. Data were still insufficient in extremely-low-birth weight infants. A significant effect was found for *Bifidobacteria* and for probiotic mixtures. Well-designed studies are warranted to clarify the choice of the most effective strain, dose and duration of supplementation before recommending use of probiotics in clinical routine [70]. Other comorbidities of prematurity, such as delayed enteral feeding, low availability of human milk [72] and immunodeficiency [73] may favour NEC occurrence. Christensen et al. [74] found that neutropenic neonates have a significant higher risk for developing NEC. Neutropenia increased with prematurity and it was more common among small for gestational age neonates. Neonates with neutropenia were treated with either recombinant granulocyte colony-stimulating factor or intravenous immunoglobulin, but unfortunately, there was no reduction in LOS or NEC [74].

Respiratory distress syndrome at birth is one of the most frequent causes of admission to the NICU. More frequently due to pulmonary disease, and rarely to an extra pulmonary cause, such as a mediastinal mass. The first case of thymic haemorrhage was described by Ribet et al. in 1971 [75]. Gargano et al. [76] described a case of thymic haemorrhage with perinatal onset, associated with bilateral haemothorax and severe respiratory distress at birth. Thymic enlargement was evident after pleural evacuation and confirmed by chest conventional radiography, computed tomography (CT) and MRI sequences. The spontaneous resolution of the enlarged thymus suggested a thymic haemorrhage that did not require surgery. Thymic haemorrhage in the perinatal period is an exceedingly rare and severe condition that often leads to stillbirth or severe respiratory distress at birth. Spontaneous thymic haemorrhage should be considered in any neonate developing acute respiratory distress with widening of the mediastinum and pleural effusion on chest radiography.

Neonatal hyperbilirubinemia is commonly due to an increase in unconjugated bilirubin that is usually benign and it resolves spontaneously. On the contrary, conjugated hyperbilirubinemia is infrequent and it should be promptly investigated. An evidence-based guideline for the management of new-borns with cholestasis [77] has been delivered this year. Early recognition of cholestasis helps to optimize the clinical management, to prevent clinical deterioration, to avoid premature, painful, expensive, and useless tests and to reduce underestimation and late referral. Many conditions can cause neonatal cholestasis and require medical or surgical treatment. The largest diagnostic group of patients includes those with biliary atresia. The clinical presentation of neonatal cholestasis may vary in relation to its aetiology. Nevertheless, the most common findings in an infant with cholestasis are prolonged jaundice, defined as jaundice lasting more than 14 days or recurring after the second week of life, acholic stools, dark yellow urine, and hepatomegaly. A practical approach for the management of the neonatal cholestasis in term and preterm infants has been provided [77]. Goetze et al. [78] recommended initial treatment and a clinically oriented overview of possible differential diagnoses.

Massage therapy is a safe practice that can promote interaction between the mother and the infant, can improve weight gain, sleep patterns, growth, development, and autonomic nervous system functions, and can reduce rates of colic and infant mortality. Some clinical studies support the use of massage for reducing neonatal jaundice [79, 80], otherwise the apparent correlation has not yet been extensively examined among neonates with jaundice who are receiving phototherapy. Lin et al. [81] investigated the effects of infant massage on newborns with jaundice who are also receiving phototherapy. A total of 56 full-term neonates with jaundice, admitted for phototherapy at a regional teaching hospital, were included in the final study and randomly assigned to the control group (29 neonates; 16 males and 13 females) and the massage group (27 neonates; 11 males and 16 females). Authors showed that by day 3 of intervention, bilirubin levels were significantly lower in the massage group while stool frequency was significantly higher.

Iron is an essential nutrient and plays a key role in many processes including human growth and development [82]. Low birth weight infants are particularly susceptible to develop iron deficiency anemia since they typically have small iron stores at birth and a greater need for iron due to the rapid increase in red cell mass [83, 84]. Nevertheless, it is currently unclear at what time iron supplementation in preterm very low birth weight infants should start. The report of Hong-Xing Jin et al. provides further insight into the issue of the timing of iron administration by evaluating early versus late iron supplementation in low birth weight infants through a meta-analysis of currently published studies [85]. Early supplementation ranged from as early as enteral feeding was tolerated to 3 weeks, and late supplementation ranged from 4 weeks to about 60 days. Early treatment was associated with significant smaller decrease in serum ferritin and haemoglobin levels (*p* < 0.001). In addition, the rate of blood transfusions was lower in early compared to late iron supplemented patients (*p* = 0.022). There was no difference between early and late supplementation in the number of patients who experienced NEC. Despite these results, authors recommended much caution when low birth weight infants are supplemented with iron as iron overload may have negative long-term effects on the neurodevelopment [86]. Neonates born with meconium stained amniotic fluid (MSAF) can develop feed intolerance in the first post-natal period of life. Gastric lavage is performed routinely in neonates with MSAF, but it is not a safe procedure as several complications have been described. The aim of Shah et al. [87] was to investigate the role of gastric lavage in vigorous late preterm and term newborns born with MSAF in a non-blinded randomized controlled trial conducted. No significant difference in the incidence of vomiting was found between 230 cases who received the gastric lavage, and 270 who did not. Feed intolerance was described in 51 neonates, with no significant difference in relation to the gestational age, gender, birth weight and modes of delivery. No complications of nasogastric tube insertion such as apnoea, bradycardia and local tissue trauma were observed in the gastric lavage group. These findings suggest that gastric lavage is not required in vigorous neonates born through MSAF to avoid feeding intolerance. Nevertheless, this topic is still debated. A systematic review of randomised controlled trials showed controversial evidences [88]. No association of feeding intolerance with gender or birth weight and gestation was found [89].

### Nephrology

Relapses and complications of nephrotic syndrome (NS), the most common manifestation of glomerular disease in childhood have been an active area of investigation. In children with steroid-sensitive nephrotic syndrome, relapses occur in most instances and 20 % to 60 % of patients develop steroid dependence. Uwaezuoke [90] reviewed triggers of relapses and their possible treatments. They documented that relapses may occur after viral infections, such as viral upper respiratory tract infections, urinary tract infections, diarrhea, or an atopic episode. This would support the hypothesis that relapses are caused by an ‘immune dysregulation’ and it is in agreement with findings of abnormalities of T cell subsets and/or T function. Rituximab a chimeric monoclonal antibody against CD 20 receptors on B cells may reduce proteinuria by inducing regulatory T cells. They also noted that relapses might be provoked by a systemic circulating factor or a primary defect of podocytes which might result in increased glomerular permeability. Corticosteroids and calcineurin inhibitors influence structure and function of podocytes. Finally, they reported that effective strategies for relapses in NS have been developed. In children, the risk for relapse has been shown to be reduced by giving prednisolone during upper respiratory infection. Zinc supplements prevent respiratory infections and reduce relapse rates in children. Ishikura et al. [91] in a long term follow-up study of 46 Japanese children with frequently relapsing NS, after an initial 2-year treatment of cyclosporine (CsA), showed that half of the patients still continued to relapse frequently or were on immunosuppressive agents at the last observation (mean age 18.7 years). They postulated that there may be an association between the experience of NS relapse during cyclosporine treatment and poorer outcome at the last observation. Fujinaga and Hirano [92] recruited 52 children with NS (median age 12.2 years). All patients had steroid-dependent NS (SDNS) or steroid-resistant NS (SRNS) and they were prescribed CsA for a median period of 39.5 months before being started on mycophenolate mofetil (MMF). During the follow-up period (median 6.1 years) after starting treatment with MMF, only 3 patients did not experience a relapse of NS, 14 were re-treated with CsA and 27 received rituximab infusions. At the last visit (median age 18.3 years), 19 patients (37 %) did not require any immunosuppressive agent, while 33 (63 %) continued to receive immunosuppressive agents. Univariate analysis revealed that the risk of persistent SDNS after MMF treatment, was positively associated with occurrence of SDNS during CsA treatment (24/33 vs. 3/19; *p* = 0.00019) and administration of rituximab during CsA treatment (12/33 vs. 0/19; *p* = 0.0017). Onset of relapse during initial CsA treatment predicts the poor clinical course of NS in the long run. The introduction of MMF following CsA treatment may not positively influence the long-term outcome in patients with SRNS and SDNS. McCaffrey et al. [93] reviewed the available evidence on complications of idiopathic NS and provided a guide for appropriate treatment. Infections should be treated promptly with broad-spectrum antibiotics in the nephrotic child. The role of antibiotic prophylaxis is still unclear, and data on the efficacy of the pneumococcal vaccine are lacking. Thromboembolic disease in NS may have potentially devastating effects, but whether primary prophylaxis is warranted is unknown. NS population are at risk for long-term cardiovascular disease and dyslipidemia, so novel management strategies, such as modifying sialylation of the circulating glycoprotein angiopoietin-like 4, have shown promising results.

### Neurology

New insights into the pathophysiology of cerebral palsy, arterial ischemic stroke and acute encephalitis may drive advances in treatment. Cerebral palsy affects about 2 of every 1000 children. Preventing strategies are needed because of lack of effective treatments. Nelson et al. [94] analysed which factors operating before labor may increase the risk of cerebral palsy. Such factors include birth defects, defined as structural or functional defects present at birth, even if they are not recognized in the newborn period. Brain defects and congenital cardiac defects are common in children born at a gestational age of at least 35 weeks with cerebral palsy. Cerebral palsy is more likely when birth defects and fetal growth restriction are combined [95]. Cerebral palsy is also associated with low gestational age at birth, probably due to disorders that lead to malformation and delivery before term. On the other hand, malformations are more likely in at term infants with cerebral palsy. Other important risk factors for cerebral palsy are marked foetal restricted growth, especially in subjects with major birth defects, intrauterine infections (cytomegalovirus), and thromboembolism in brain, other organs and placenta. Thromboembolism may lead to malformations and perinatal stroke, the most common cause of cerebral palsy in children born at term. Finally, it has been found that a genetic component [96] is involved in many cases of cerebral palsy, especially when an older sibling is affected. A prevention programme should be based on early investigation of key prenatal risk factors for cerebral palsy, including birth defects, fetal growth restriction, and neonatal encephalopathy, that may act directly or may co-operate with other exposures or intrapartum events such asphyxia.

In childhood, stroke is rare but the incidence of arterial ischemic stroke (AIS) in the neonate is similar to the incidence of large artery AIS in adults and is 17 times greater than the incidence of AIS in children. AIS is associated with a significant morbidity and mortality. In term neonates, AIS is the most common cause of cerebral palsy and is the second most common of seizures. Therefore, challenges presented by children with stroke have been the subject of considerable interest. Mechanisms, diagnosis and management of AIS in children have been summarized by Rosa et al. [97]. They documented that factors associated with AIS in childhood differ from those in adults. Focal cerebral arteriopathy caused by infections or genetically determined (Moyamoya Syndrome) seems to be the main cause. Other factors involved in the occurrence of AIS are vasculitis, arteriovenous malformation or dysplasia, hereditary coagulopathies, hematologic disorders, SCA, infarctions due to metabolic diseases, head or neck trauma, congenital and acquired heart disease. They pointed out that clinical presentation of stroke depends on the involved artery and patient’s age. Seizures and headache may develop irrespective of stroke subtype. Infants with stroke often present aspecific manifestations. Seizures and altered mental status may be the only symptoms. They emphasized that the cornerstone for diagnosing paediatric stroke is neuroimaging. In emergency settings, the diagnostic importance of non-contrast Computer Tomography has been underlined. However, MRI remains the “gold standard”. MRI Angiography should be useful to assess the involved territory. Vascular imaging of the carotid and vertebral arteries by duplex ultrasonography, Computer Tomography angiography, and catheter angiography should be considered. Laboratory tests for the investigation of diseases associated with AIS in infants and children should be performed.

Understanding of the underlying etiology of AIS provides a basis for choosing the best therapeutic options. After excluding a haemorrhagic stroke, anticoagulant therapy is mandatory using aspirin or heparin. Thrombolysis may be considered. Further episodes of AIS may be prevented by Acetil-salycilic acid. Low molecular-weight heparin or warfarin are useful in children with cardioembolism, extracranial arterial dissection, cerebral venous sinus thrombosis and when aspirin treatment fails. Rosa et al. acknowledge that further studies are warranted to optimize antithrombotic treatment. Control of associated diseases, such as SCA or metabolic diseases is also recommended for secondary stroke prevention. Finally, rehabilitation can reduce long-term morbidity and improve quality of life.

Encephalitis is a serious and disabling condition that can result from a large number of causes even if many cases remain undiagnosed. In a case series of acute encephalitis, Pillai et al. [98] provides insights into the relative frequency of causes. They observed that 49 (30 %) of 164 children had an infectious encephalitis, 13 (8 %) children had an infection associate encephalopathy. Pathogens detected in infectious encephalitis were enterovirus (12), mycoplasma pneumoniae (7), herpes simplex virus (5), and cytomegalovirus 2 %, in infection-associated encephalopathy, influenza virus and rotavirus. Immune-mediated/autoantibody-associated encephalitis was diagnosed in 56 (34 %) children. It was due to acute disseminated encephalomyelitis in 21 % of cases, N-methyl-D-aspartate receptor antibody encephalitis in 6 % of cases, voltage-gated potassium channel complex antibody encephalitis in 4 % of cases. In 46 (28 %) patients, the cause of encephalitis was unknown. Young children were more frequently affected with a median age at presentation of 5.5 years. The peak was during the winter season. Risk factors for poor outcomes were intensive care unit admission, diffuse restriction on MRI and status epilepticus.

### Nutrition

This year saw the publication of the position statement of the Task Force on Breastfeeding of the Ministry of Health, the Italian Society of Neonatology, the Italian Society of Primary Pediatric Care and the Italian Society of Pediatric Gastroenterology and Nutrition [99]. They pointed out that breastfeeding is of benefit to the mother and the infant and should be promoted not only by neonatologists and pediatricians, but also by professionals, especially in maternity hospitals. They recommended exclusive breastfeeding for about 6 months of life. They emphasized that poor growth may suggest anticipating the introduction of weaning foods after 4 months of age. In agreement with those recommendation, there is evidence that starting breastfeeding within the first hour of life was associated with a reduced risk of neonatal mortality [100]. Moreover, exclusively breastfed newborns had a lower risk of mortality and infection-related deaths in the first month than partially breastfed neonates. Exclusively breastfed newborns also had a significant lower risk of sepsis, diarrhea and respiratory infections compared with those partially breastfed [100]. Khan et al. [100] concluded that substantial benefits in reducing neonatal mortality and morbidity can be achieved with effective promotion of early initiation of breastfeeding and exclusive breastfeeding during the first month of life.

Giugliani et al. [101] reviewed studies that evaluated the effect of any type of intervention for promoting breastfeeding on child weight, length (or height) and weight/height (or BMI). Meta-analyses of studies reporting on mean weight, length, weight/length or BMI showed that the interventions had no impact on weight or length/height z scores and had a modest, but significant, reduction in body mass index/weight-for-height z scores, which was limited to studies from low- and high-incomes settings. An important heterogeneity among studies should be taken into account when interpreting the results. Authors stated that it is not possible to have a conclusive analysis of the impact of breastfeeding promotion interventions on child growth, although these seem to have little influence on child growth. Nevertheless, breastfeeding promotion must remain a priority, as many other benefits to the child and mother are already well established.

Breast milk provides both protective and stimulatory immune signals, which may confer lower susceptibility to disorders such as allergic disease. Lodge et al. [102] systematically review the association between breastfeeding and childhood allergic disease. Longer duration of breastfeeding was associated with reduced risk of asthma for children (5–18 years), particularly in medium-/low-income countries, but this estimate had high heterogeneity and low quality. Exclusive breastfeeding for 3–4 months was associated with reduced risk of eczema ≤2 years (estimate principally from cross-sectional studies of low methodological quality). No association was found between breastfeeding and food allergy (estimate had high heterogeneity and low quality).

The recommendations concerning complementary feeding from the Italian Society of Gastroenterology, Hepathology and Pediatric Nutrition and the Italian Society of Pediatric Allergy and Immunology Emilia Romagna [103] have been issued. Solid foods should be introduced between 4 and 6 months of age when the 6 months limit is not possible. An early exposure (before 4 months of age) or a delayed introduction of the main allergenic foods (after 6 months of age) has not been found to be useful to prevent allergy, even if infants were at risk of atopy. They noted that weaning while continuing breastfeeding might reduce the onset of allergies. The timing of introduction and amount of gluten has no effect on the onset of coeliac disease. The timing of introduction of complementary feeding is not related with the risk of obesity, T2DM and cardiovascular disease in later ages. They emphasized that infants should go on a nutritionally balanced diet, encouraging the daily consumption of fruit and vegetables. They recommended providing parents with the right information about daily intake of carbohydrates, lipids, proteins, liquids, fibers, vitamins and minerals. Baby led weaning and auto-weaning were discussed as a promising way for introducing complementary foods which respects the child’s self-regulatory capacity. They point out that baby led weaning is feasible when infants achieve postural stability to sit and to grasp objects.

Zheng et al. [104] emphasized the risk of early weaning. They investigated the association between timing and types of complementary feeding and adiposity in children of 4–5 years of age enrolled in the Jiaxing birth cohort. Among 40510 children, 3.18 were overweight and 64.8 % were fed complementary food before 3 months of age. Early introduction of complementary foods before 3 months of age, was associated with greater BMI z-score (P-trend < .001) and higher risk of overweight (P-trend = .033). No significant association between timing of complementary feeding and obesity was observed. Fish liver oil was the major type of complementary food associated with adiposity when it was introduced before 3 months of age.

Nutrition in early life is increasingly considered to be an important factor influencing later health. There is evidence that foods rich in n-3 polyunsaturated fatty acids have protective effects on oxidative damage and inflammation of the lung tissue, as in childhood wheezing and asthma. Lumia et al. [105] explored in a birth cohort study the association of food consumption in early life and the risk of atopic and non-atopic asthma. Among 182 children with asthma and 728 controls, a higher consumption of cow’s milk products was inversely associated with the risk of atopic asthma and higher consumption of breast milk and oats inversely with the risk of non-atopic asthma. Early consumption of fish was associated with a decreased risk of all asthma. This study indicated that dietary intake in early life combined with atopy history has an impact on the risk of developing asthma.

A diet rich in fruits and vegetables supports healthy weight and weight loss in the context of a reduced-caloric diet. Moreover, it protects against chronic disease such as diabetes, stroke, cancer, and all-cause mortality. Herrick et al. [106] described youth fruit consumption, using data from 3129 participants, aged 2 to 19 years, in the US National Health and Nutrition Examination Survey. They found that 53 of total fruit intake was consumed as whole fruits and 34 % as fruit juices. Apples, apple juice, citrus juice, and bananas were responsible for almost half of total fruit consumption. There were differences by race and Hispanic origin in intake of citrus fruits, berries, melons, dried fruit, and citrus juices and other fruit juices. Children aged 6 to 11 years, consumed more apples and less bananas and other fruit juices compared with youth aged 2 to 5 years. Variations in the type of fruits consumed may be related to taste preference, repeated exposures to fruits, social experiences, and availability.

Augusto et al. [107] underlined the importance of effective actions to promote the consumption of fruits and vegetables. They studied the relationship between frequency of fruit and vegetable consumption and nutritional deficiencies in 702 Brazilian schoolchildren (aged 4–10 years) in a cross-sectional population-based study. Only 5 % of children consumed fruits and vegetables ≥5 times/day. Overall, 6.3 of children were anaemic, 3.3 were stunted, 2.7 were obese and 33 % had multiple nutritional deficiencies. Consumption of vegetables ≤3 times/month and of fruits ≤3 times/week was associated with lower plasma concentrations of carotenoids and vitamin E. Nutritional deficiencies, including anemia, vitamin E insufficiency, vitamin D insufficiency and stunting, were more common in non-consumers of fruits and vegetables than in usual consumers (vegetables ≥1 time/week and fruits ≥4 times/week).

Trends in childhood morbidity and mortality are strongly correlated with childhood infections and undernutrition. Most of the morbidity and mortality from diarrhea and pneumonia occur in children under 2 years of age, linking it closely to early nutrition and growth. Because of the complex interrelation between infections and nutrition conditions, Salam et al. [108] addressed several strategies to reduce major childhood infections and improve nutrition and growth and implications. These interventions include the following: exclusive breastfeeding up to 6 months of age, promotion of complementary feeding, pneumococcal, rotavirus and *Haemophilus influenzae type b* vaccine, community-based management of neonatal infections, case management of pneumonia infections, use of oral rehydration salt and zinc in diarrhea, vitamin A supplementation, antibiotics for dysentery, and Water Sanitation and Hygiene strategies. Utilization of preventive interventions will make an impact by reducing malnutrition and under-5 morbidity and mortality.

White et al. [109] measured the prevalence of malnutrition, obesity and nutritional risk in 832 pediatric inpatients among multiple hospitals throughout Australia in a single day. The prevalence rates of malnourished, wasted, stunted, overweight and obese pediatric patients were 15, 13.8, 11.9, 8.8 and 9.9 %, respectively. Aboriginal and Torres Strait Islander patients were more likely to have lower height-for-age z-scores (*P* <0.01). However, BMI and weight-for-age z-scores were not significantly different. Children who were younger, from regional hospitals or with a primary diagnosis of cardiac disease or cystic fibrosis had significantly lower anthropometric z-scores (*P* = 0.05). Forty-four % of patients were identified as at high nutritional risk and requiring further nutritional assessment. They concluded that malnutrition and nutritional risk of Australian pediatric inpatients on a given day was much higher when compared with the healthy population. In contrast, the proportion of overweight and obese patients was less.

### Oncology

Survival after childhood cancer is greatly enhanced in last decades. PanCare, an interdisciplinary Network of experts in the field, has been created to identify best practices of long-term care and address challenges in survivors, especially related to late effects of treatment [110]. Rhabdomyosarcoma (RMS), the most frequent soft tissue sarcoma in childhood, can be distinguished in embryonal RMS which occurs in 75 % of cases, with a higher incidence at age of 0–4 years and alveolar RMS found at every age with a worse prognosis. RMS has also been classified basing on molecular prognostic markers. PAX–FOXO1 fusion gene status was identified and compared to alveolar histology, whereas fusion gene–negative alveolar RMS patients were clinically similar to ERMS patients. Attempts to identify other prognostic gene signatures an independent 5-gene signature, MG5, showed a significant association with overall survival in the fusion gene–negative alveolar RMS and embryonal RMS patients. Hingorani et al. [111] studied whether expression of the MG5 metagene, measured using a technical platform that can be applied to routine pathology material, would correlate with outcome in a cohort of children with fusion gene–negative alveolar RMS. MG5 signature score showed a significant correlation with overall and failure-free survival. The ability of MG5 signature was confirmed to identify different risk groups within fusion gene–negative alveolar RMS from a different patient cohort composed of those with intermediate-risk disease. Ma et al. [112] explored the treatment results of childhood RMS and identified prognostic factors in a multicenter Chinese retrospective study. Medical records of 161 patients (median age 51 months) were analyzed. The genitourinary system was the most common primary site of tumor (43.5 %). The histological findings were: 80.7 embryonal, 11.9 alveolar and 3.1 % botryoid type. The 10-year event free survival rate was 53.4 ± 5.1 %, overall survival was 65.3 ± 6.3 %. Among variables, age (*p* = 0.028) and disease groups (*p* = 0.000) were significantly associated with overall survival. In conclusion, epidemiological characteristics of patients of this study were similar to worldwide data.

Sangkhathat et al. [113] described current management of pediatric soft tissue sarcomas, based on multidisciplinary approach including surgery, chemotherapy and radiation therapy. Decision making in management protocol for each patient is determined on the risk induced by various clinical and pathological parameters. For cases of small resectable tumors in a favorable site, surgery provides the best choice of local control. Radiation therapy is added when surgery leaves residual disease or there is evidence of regional spread. Chemotherapy reduces risks of relapse and improves overall survival. The standard therapy for pediatric RMS is a multi-agent chemotherapy and surgery resection. This line aims to reduce the aggressiveness of the required surgery and frequently helps preserve organ function. Radiotherapy is often added. Proton beam therapy (PBT) is a type of radiotherapy with excellent dose localization. Takizawa et al. [114] showed that PBT can be useful in cases who cannot receive definitive photon radiotherapy. They performed, safely and effectively, fPBT in a 1-year-old girl with alveolar RMS with liver and cardiac invasion at the dose of 41.4 GyE. In this case, chemotherapy alone was not effective; surgery was not possible for broad tumour invasion of the surrounding diaphragm; photon radiotherapy could not be performed because the liver could not tolerate the treatment dose. In a retrospective study, Fukushima et al. [115] analyzed five children with genitourinary/pelvic rhabdomyosarcoma (GU/P-RMS) who had undergone multimodal therapy combined with PBT in a Japanese institution. All received neo-adjuvant chemotherapy and 3 underwent chemotherapy during PBT (Group Cx). All patients of Group Cx developed leukocytopenia but survived by their last hospital visit (after 36 months). They concluded that PBT was well tolerated and could be a plausible choice instead of photon therapy for GU/P-RMS children.

### Pulmonology

Deletions of or mutations in genes encoding proteins important in surfactant production and function (SP-B, SP-C, and ABCA3), surfactant catabolism (GM-CSF receptor), or transcription factors important for surfactant production (TTF1) or lung development (Fox F1) may cause pediatric diffuse lung disease [116]. This is an heterogeneous group of uncommon disorders with impaired gas exchange and diffuse infiltrates at chest imaging [117]. Montella et al. [118] report on a 3-years-old boy with dry cough, progressive hypoxemia, dyspnea and bilateral ground glass opacities at chest high-resolution computed tomography (HRCT), in whom lung histology strongly suggested a surfactant disorder, but no variants conclusively classified as pathogenic in genes encoding surfactant proteins or transcription factors important for surfactant production were found. Pulse intravenous high-dose methylprednisolone, oral hydroxychloroquine and azithromycin were started as treatment, leading to gradual weaning from oxygen and to improvement of cough and dyspnea. Six months later, steroids and oxygen were stopped and a substantial improvement of exercise tolerance versus pre-treatment test was observed. Currently, the subject is 6 years-old, adherence to treatment is satisfactory, with no side effects. Repeated daily oxygen transcutaneous saturation showed values not lower than 98 % at room air, with median overnight values of 96 % (min 74 %, max 99 %), while chest HRCT was unmodified.

The authors confirm that in children with suspected surfactant dysfunction, genetic testing is strongly recommended because it can provide a definitive diagnosis but when causative mutations are not found, lung biopsy with consistent histology may help physicians to address the definitive diagnosis.

Respiratory Syncytial Virus (RSV) is by far the pathogen responsible for most of the airway infections in children under age 2 years. RSV bronchiolitis is a leading cause of severe respiratory disease that requires hospitalization and, in some cases, intensive care [119]. Quinonez et al. [120] pointed out important issues in the management of bronchiolitis, Pulse oximetry potentially overdiagnoses hypoxemia and increase hospital admission and length of stay [121]. Another point is that infants with bronchiolitis should be provided with antipyretics, intravenous hydration, oxygen when necessary, and salbutamol should be given to assess its efficacy. Finally, continuous treatment with epinephrine or saline may increase length of hospital stay and oxygen requirement when compared with “on-demand” treatments [122]. Infection can be consistently prevented by the application of environmental, hygienic and sanitary measures to minimize the viral diffusion [123]. Recommendations drafted by the Italian Society of Neonatology concerning prophylaxis of RSV infection with palivizumab, a humanized monoclonal antibody that blocks the viral replication during the epidemic season to children at high risk have been revised in 2015 through the update of epidemiological data [124]. Lanari et al. [125] evaluated the risk factors for hospitalization due to bronchiolitis during the first year of life in children born at different gestational ages in Italy. The study population consisted of 2314 newborns, of which 2210 (95.5 %) had a 1 year follow-up and were included in the analysis, while the remaining 120 cases (5.4 %) were hospitalized during the first year of life for bronchiolitis. Children born at 33–34 weeks gestational age had a higher hospitalization rate compared to other groups. Male gender, prenatal exposure to maternal smoking, neonatal surfactant therapy, having siblings aged less than 10 years, living in crowded conditions and being exposed to epidemic season during the first 3 months of life were identified as risk factors for high rate of hospitalization for bronchiolitis. This confirms that individual characteristics and exposure to environmental factors play an important role in determining the risk of severe infection and hospitalization, independently from preterm birth. The analysis of the weight of each risk factor allows defining accurately the risk for bronchiolitis hospitalization for any infant during the first year of life and letting authors to conclude that indications of palivizumab should be individually “tailored”.

## Conclusions

This was an exciting year for pediatric research. This review provides novel information with regard to pathophysiology, clinical aspects, prevention and management of common diseases in childhood. Several Position statement of scientific societies on different issues including nutrition and hyperbilirubinemia in newborns were made available. New data on rare diseases, such as disorder of surfactant metabolism, and 48,XXXY/49,XXXXY mosaicism may improve their understanding. Overall, these advances can improve diagnosis and treatment of pediatric diseases and offer comprehensions on how we can develop patient care in the future.

## Abbreviations

AIS: Arterial Ischemic Stroke
AOM: Acute Otitis Media
BMI: Body Mass Index
CD: Celiac Disease
CsA: Cyclosporine
CT: Computed Tomography
EIB: Exercise Induced Bronchoconstriction
FeNO: Fractional Exhaled Nitric Oxide
GD: Graves’ Disease
HRCT: High-Resolution Computed Tomography
HT: Hashimoto’s Thyroiditis
ID: Invasive Disease
KS: Klinefelter Syndrome
LOS: Late-Onset Neonatal Sepsis
MMF: Mycophenolate Mofetil
MRI: Magnetic Resonance Imaging
MSAF: Meconium Stained Amniotic Fluid
NEC: Necrotizing Enterocolitis
NICU: Neonatal Intensive Care Units
NS: Nephrotic Syndrome
PBT: Proton Beam Therapy
P-SEP: Presepsin
PWS: Prader Willi Syndrome
rGH: Recombinant Growth Hormone
rIGF-1: Insulin-Like Growth Factor-1
RMS: Rhabdomyosarcoma
RSTS: Rubinstein-Taybi Syndrome
RSV: Respiratory Syncytial Virus
SCA: Sickle-Cell Anemia
T2DM: Type 2 Diabetes Mellitus.
